# Carbon Monoxide Poisoning on a Motor Launch

**Published:** 1910-12-17

**Authors:** 


### Toxicology.

### CARBON MONOXIDE POISONING ON A MOTOR LAUNCH.

At the present time the explosive engine burning petrol or allied liquid is in extensive and increasing use, especially, perhaps, as providing a cheap and convenient means of propulsion. A device that rendered possible the motor-car and the motor-boat, not to speak of the submarine and the aeroplane, must be accounted a signal boon to mankind. But, unfortunately, like every good gift, the explosive engine is not perfect, and has certain disadvantages and dangers. One of these is illustrated by the following case:—

On April 14 last five men went out fishing on a motor-launch propelled by a 6-h.p. benzine engine. Of the five men on board four were regular fishermen and one a friend having a day's outing. This man was a strong healthy young fellow, about 20 years of age; he was in good health, and apparently enjoyed his holiday. When darkness came on fishing ceased, and the launch started on the return journey. The regular crew set to work as usual, one going into the cabin to mind the engine, the others remaining outside and occupying themselves with the fish. The visitor feeling drowsy, and having no particular task, went into the cabin, crawled right forward past the engine, and lay down to sleep. This was about 8 p.m. Shortly after, the man tending the engine heard the visitor groaning, and called out to him, but receiving no reply thought he was perhaps feeling sea-sick in the heat of the cabin, and wished to be left alone. Apart from this the visitor lay quiet, and was presently thought to be asleep. About 11.30 p.m. the boat arrived home, and as the visitor did not turn out one of the crew went to wake him. This, however, was found impossible; he could not be roused, and assistance was sent for. Dr. Barclay, who records the case in the *New Zealand Medical Journal*, vol. vii., No. 30, saw him about midnight. He had been removed to a neighbouring house and was lying on a couch. The face looked natural in colour, so much so that at first glance Dr. Barclay thought the man was living, and was surprised on examination to find that he was dead. The body was still warm, but no sign of heart's action or of breathing could be noticed, even after hypodermic injection of strychnine and prolonged use of artificial respiration.

Next day a post-mortem examination was made, and the following facts were noted: (1) The hypostatic lividity was rather more marked than usual, and the colour distinctly red; the lips were pinkish red, the face looking much as if life were present. (2) On making the first incision over the sternum, blood flowed out freely, collecting in the hollows above the clavicle; the colour of the blood was peculiar, resembling most nearly that of vermilion; the same unusually fluid, vermilion-coloured blood was found in all parts of the body—*e.g.* after removing the skull-cap blood escaped freely for a time, then dripped slowly for hours; the inner coating of the stomach, the surface of the lungs showed in places a distinct pink colour; the kidneys, liver, and especially the brain were congested. Otherwise no abnormalities were revealed, and deceased had evidently been a strong, healthy young man. Having thus excluded organic disease, the suddenness of death, the external appearance of the body, and more especially the colour of the blood as found at the post-mortem examination, suggested poisoning by CO. Benzine vapour was suggested as a possible toxic agent. Without discussing this further, a sample of the vermilion-coloured blood from this body was sent to Dr. McLaurin, Government Analyst, Wellington. He reported that CO was present, the blood being about 50 per cent. saturated. Thus it seems clear that in this case the cause of death was carbon monoxide. Further, as there was no coke fire or other possible source of this gas on board the launch, it may be taken as proved that the CO was derived from the benzine engine in use.

Upon further inquiry it was found that it is not unusual for the men attending to these engines in the cabins of launches to experience toxic symptoms of a lesser degree—*e.g.* headache and dizziness. Thus the engineer of this launch stated that on the same night as the fatality occurred he himself "felt queer," but was able to continue at work till the boat arrived home. He was able to help with the corpse, and rode off on a bicycle for assistance, but on returning he partially collapsed. Dr. Barclay found him sitting in the wet grass against a fence, feeling faint and with a feeble pulse. He had to be helped indoors, but recovered with rest and stimulation. This collapse, some time after coming into the fresh air, is like what is experienced in CO poisoning. The difference in degree of poisoning is probably explained by the fact that the visitor was sleeping in the very forepart of the cabin, in a recess extending under the deck and without any opening for ventilation, whilst the engineer was sitting at the back of the cabin, receiving the benefit from an open port-hole fixed half-way forward in the cabin. Thus the visitor lay in comparatively stagnant air; the engineer sat in the best ventilated part of the cabin.

Carbon monoxide is a particularly dangerous gas. When mixed with air it has no warning smell, and it is easily breathed; hence its action is insidious. And it is also very deadly, depending on the essential fact that CO when inhaled forms with the hæmoglobin of the blood a fairly staple compound—carbon-monoxide-hæmoglobin—which is useless for the tissues of the body and cannot easily be broken up, for example, by a free supply of oxygen or fresh air. This carbon-monoxide-hæmoglobin gives the distinctive cherry colour to the blood and tissues. A very small percentage of CO in the air breathed will produce fatal results. Hence a comparatively small escape of the products of combustion from the engine would suffice to render dangerous the air of the engine room. The carbon monoxide would be removed in part by ventilation, but it would tend to accumulate in the undisturbed air of the recess where the visitor lay. In these launches the exhaust pipe opens outside the boat, and apparently there is not much danger of leakage from any part of the exhaust. But there is always some escape of products of combustion from the cylinders of these engines; increased probably by worn or badly adjusted cylinder fittings, increased, perhaps, and certainly made more dangerous, when owing to the imperfect adjustment of the supply of air and benzine, the combustion of the latter is incomplete. Thus it is when the engine is "firing badly" that an offensive smell is most likely to be noticed. To obtain protection from sea and rain these engines must be under cover. Unfortunately, if this be overdone and no proper ventilation provided, any CO given off by the engine in the confined space of the cabin may soon reach a dangerous amount. Compare the fact that most cases of poisoning by the fumes of motor-cars have occurred when the patient has been working the engine of the car in a garage or other closed space.

Evidently there is, under certain conditions, a very real danger to the numerous users of these motor launches. It is important that they be warned, in order that precautions may be taken to prevent any further accidents. With this object Dr. Barclay ventures to suggest the following measures to be observed on motor-boats:—

1. Adjust the supply of benzine and air as accurately as possible to secure complete combustion.
2. See that cylinder fittings are not leaking unduly.
3. Ensure as much ventilation as possible in the engine-room.
4. Whenever possible, sleeping quarters should be entirely and efficiently separated from the engine-room.

